# Supplementary material for: Single Cell Transcriptomic and Chromatin Profiles Suggest Layer Vb Is the Only Layer With Shared Excitatory Cell Types in the Medial and Lateral Entorhinal Cortex
Source: Front Neural Circuits. 2022 Jan 26;15:806154. doi: 10.3389/fncir.2021.806154 (PMC8826650; doi:10.3389/fncir.2021.806154)
Supplement: Supplementary file 14 [file Data_Sheet_1.ZIP › LEC_Sup_vs_MEC_Sup_Motifs/homerResults/motif3.similar.html]

motif3

## Information for motif3

G
T
C
A
G
C
A
T
A
C
G
T
C
G
T
A
T
C
G
A
C
G
A
T
G
T
A
C
C
G
T
A
C
A
T
G
G
T
A
C
  
Reverse Opposite:  

C
A
T
G
G
T
A
C
C
G
A
T
C
A
T
G
C
G
T
A
A
G
C
T
G
C
A
T
T
G
C
A
C
G
T
A
C
A
G
T
  

|  |  |
| --- | --- |
| p-value: | 1e-74 |
| log p-value: | -1.715e+02 |
| Information Content per bp: | 1.640 |
| Number of Target Sequences with motif | 4209.0 |
| Percentage of Target Sequences with motif | 31.57% |
| Number of Background Sequences with motif | 4890.5 |
| Percentage of Background Sequences with motif | 24.54% |
| Average Position of motif in Targets | 302.6 +/- 229.5bp |
| Average Position of motif in Background | 350.9 +/- 277.7bp |
| Strand Bias (log2 ratio + to - strand density) | 0.0 |
| Multiplicity (# of sites on avg that occur together) | 1.30 |
| Motif File: | file (matrix) reverse opposite |

### Similar de novo motifs found

|  |  |  |  |  |  |  |  |
| --- | --- | --- | --- | --- | --- | --- | --- |
| Rank | Match Score | Redundant Motif | P-value | log P-value | % of Targets | % of Background | Motif file |
| 1 | 0.936 | G C A T T C A G C G T A A G C T A C G T G T C A G C T A C A G T | 1e-63 | -145.683084 | 41.01% | 34.00% | motif file (matrix) |
| 2 | 0.673 | G A T C C T A G T G A C G A C T T C G A G C T A G A C T G C A T | 1e-59 | -138.015117 | 51.66% | 44.59% | motif file (matrix) |
| 3 | 0.760 | C G T A C G A T C G A T C G T A T G C A A C G T C A T G C G T A T A G C A C G T T G A C C G T A | 1e-51 | -118.662189 | 19.96% | 15.07% | motif file (matrix) |
| 4 | 0.631 | G C T A A T C G G C A T A C T G G T C A C G A T T G C A G C T A G C A T C A G T C T G A C G A T | 1e-48 | -112.529187 | 54.64% | 48.25% | motif file (matrix) |
| 5 | 0.691 | C G A T C T A G C G T A T A C G A G C T T A G C G T C A A G T C C T A G T A C G | 1e-38 | -88.797565 | 31.19% | 26.12% | motif file (matrix) |
| 6 | 0.706 | C G T A C G T A A C G T A C G T C G T A C G T A A C G T C G T A C G T A A G T C C G T A A G T C | 1e-25 | -59.537910 | 10.84% | 8.20% | motif file (matrix) |
| 7 | 0.648 | C G T A T A G C C G T A C A T G G A C T C T G A C G T A C G A T G C T A C G T A G C A T T G A C | 1e-22 | -51.427272 | 21.41% | 18.06% | motif file (matrix) |
| 8 | 0.636 | G A C T G C A T C A G T G C A T A C G T C G T A G C A T A C G T | 1e-6 | -14.719873 | 54.70% | 52.57% | motif file (matrix) |
| 9 | 0.629 | C G A T C G T A C G T A C G A T A G C T C G A T T G C A G A T C | 1e-4 | -11.127993 | 20.34% | 18.91% | motif file (matrix) |
| 10 | 0.620 | T G C A A C G T C G T A C T G A C G T A C G A T C G A T C G T A | 1e-3 | -7.001648 | 32.64% | 31.38% | motif file (matrix) |
| 11 | 0.659 | C G T A A C T G C G T A A C T G A C G T C G T A C G T A A C G T C G T A C G T A | 1e-2 | -4.771816 | 0.24% | 0.15% | motif file (matrix) |
| 12 | 0.648 | C G T A A C G T A C G T C G T A A C G T A G C T A C T G C T G A | 1e-1 | -4.430358 | 2.32% | 2.04% | motif file (matrix) |
